# Global burden of viral infectious diseases of poverty based on Global Burden of Diseases Study 2021

**DOI:** 10.1186/s40249-024-01234-z

**Published:** 2024-10-08

**Authors:** Xin-Chen Li, Yan-Yan Zhang, Qi-Yu Zhang, Jing-Shu Liu, Jin-Jun Ran, Le-Fei Han, Xiao-Xi Zhang

**Affiliations:** 1 School of Global Health, Chinese Center for Tropical Diseases Research, Shanghai Jiao Tong University School of Medicine, Shanghai, People’s Republic of China; 2 Institute of One Health, Shanghai Jiao Tong University, Shanghai, People’s Republic of China; 3 School of Public Health, Shanghai Jiao Tong University School of Medicine, Shanghai, People’s Republic of China

**Keywords:** Viral infectious diseases of poverty, Global Burden of Disease 2021, Disability-adjusted life years, Socio-Demographic Index

## Abstract

**Background:**

Viral infectious diseases of poverty (vIDPs) remain a significant global health challenge. Despite their profound impact, the burden of these diseases is not comprehensively quantified. This study aims to analyze the global burden of six major vIDPs, including coronavirus disease 2019 (COVID-19), HIV/AIDS, acute hepatitis, dengue, rabies, and Ebola virus disease (EVD), using data from the Global Burden of Diseases, Injuries, and Risk Factors Study 2021 (GBD 2021).

**Methods:**

Following the GBD 2021 framework, we analyzed the incidence, mortality, and disability-adjusted life years (DALYs) of the six vIDPs across 204 countries and territories from 1990 to 2021. We examined the association between the Socio-Demographic Index (SDI) and the burden of vIDPs. All estimates were reported as numbers and rates per 100,000 population, calculated using the Bayesian statistical model employed by GBD 2021, with 95% uncertainty intervals (UI).

**Results:**

In 2021, vIDPs caused approximately 8.7 million deaths and 259.2 million DALYs, accounting for 12.8% and 9.0% of the global all-cause totals, respectively. Globally, the burden of vIDPs varied significantly: COVID-19 caused around 7.9 million (95% UI: 7.5, 8.4) deaths and 212.0 million (95% UI 197.9, 234.7) DALYs in 2021. Acute hepatitis had the second-highest age-standardized incidence rate, with 3411.5 (95% UI: 3201.8, 3631.3) per 100,000 population, while HIV/AIDS had a high age-standardized prevalence rate, with 483.1 (95% UI: 459.0, 511.4) per 100,000 population. Dengue incidence cases rose from 26.5 million (95% UI: 3.9, 51.9) in 1990 to 59.0 million (95% UI: 15.5, 106.9) in 2021. Rabies, although reduced in prevalence, continued to pose a significant mortality risk. EVD had the lowest overall burden but significant outbreak impacts. Age-standardized DALY rates for vIDPs were significantly negatively correlated with SDI: acute hepatitis (r = −0.8, *P* < 0.0001), rabies (r = −0.7, *P* < 0.0001), HIV/AIDS (r = −0.6, *P* < 0.0001), COVID-19 (r = −0.5, *P* < 0.0001), dengue (r = −0.4, *P* < 0.0001), and EVD (r = −0.2, *P* < 0.005).

**Conclusions:**

VIDPs pose major public health challenges worldwide, with significant regional, age, and gender disparities. The results underscore the need for targeted interventions and international cooperation to mitigate the burden of these diseases. Policymakers can use these findings to implement cost-effective interventions and improve health outcomes, particularly in regions with high or increasing burdens.

## Background

In the twenty-first century, global health faces numerous challenges, with infectious diseases remaining a major source of the global health burden. According to Global Burden of Disease 2019 (GBD 2019) data, infectious diseases continue to be a leading cause of mortality and morbidity, particularly among children and the elderly in low- and middle-income countries [1]. Despite significant advancements in global public health governance over the past few decades, which have led to the effective control and prevention of many infectious diseases, emerging and re-emerging infectious diseases still pose a constant threat to global health security. For instance, the Ebola virus disease (EVD) outbreak of 2014–2016 and coronavirus disease 2019 (COVID-19) pandemic that began in 2019 both highlighted the vulnerability of global public health systems and the urgent need for robust responses to infectious diseases [2].

Among various infectious diseases, viral infections are of particular concern to global public health due to their high transmissibility, rapid spread, and significant threat to public health [3]. Currently, extensive research efforts are dedicated to exploring the transmission mechanisms, clinical manifestations, and prevention strategies of viral infections. However, despite significant advancements in the study of viral infections, viral infectious diseases of poverty (vIDPs) such as HIV/AIDS, acute hepatitis, dengue fever, rabies, and EVD often do not receive the same level of comprehensive evaluation and systematic analysis as more globally prominent diseases like COVID-19. Considering factors such as global impact, data availability, and research representativeness, and based on the types of vIDPs mentioned in Zhou's report [4], we primarily focus on six vIDPs, including COVID-19, AIDS, acute hepatitis, dengue fever, rabies, and EVD. COVID-19, caused by the novel coronavirus (SARS-CoV-2), has rapidly spread worldwide since its outbreak in late 2019, resulting in millions of cases and deaths, and severely impacting global economies and social life [5]. HIV/AIDS, a chronic infectious disease caused by the human immunodeficiency virus (HIV), compromises the immune system, making individuals more susceptible to other infections and diseases [6]. HIV/AIDS remains widespread globally, with particularly high infection and mortality rates in sub-Saharan Africa [7]. Acute hepatitis, including hepatitis A, B, C, and E, is primarily transmitted through different routes: hepatitis A and E are mainly spread through contaminated food and water; hepatitis B and C are mainly spread through blood and bodily fluids. These viral infections can lead to liver inflammation and damage [8]. Acute hepatitis is prevalent worldwide, especially in areas with poor sanitary conditions, posing a significant threat to liver health and overall public health [9]. Dengue fever is a mosquito-borne acute infectious disease prevalent in tropical and subtropical regions [10]. It results in millions of cases annually, placing immense pressure on local public health systems. Rabies is a fatal infectious disease transmitted through the bites of infected animals [11]. Despite being preventable through vaccination, rabies still causes tens of thousands of deaths each year, primarily in developing countries, particularly in rural areas [7]. EVD is a severe acute infectious disease with a high fatality rate, transmitted through contact with the bodily fluids of infected individuals [12]. Although EVD outbreaks are relatively infrequent, each outbreak severely impacts local public health systems, especially in certain regions of Africa [7].

The burden of vIDP constitutes a significant component of the global disease burden. These diseases not only result in substantial mortality but also pose major challenges to healthcare systems, economic development, and social stability. The high incidence and mortality rates of AIDS in sub-Saharan Africa markedly increase the disease burden in this region. Viral infectious diseases also place immense pressure on healthcare systems. Outbreaks of dengue fever in tropical and subtropical regions lead to a large number of hospital admissions, significantly straining local healthcare infrastructure. Furthermore, viral infectious diseases have a profound negative impact on the global economy. The COVID-19 pandemic has drastically reduced global economic activity, leading to increased unemployment rates and substantial economic losses in many countries [13]. These diseases also have far-reaching effects on social stability and mental health. The outbreak of EVD triggered widespread social panic and discrimination, undermining community cohesion and social trust. Quarantine measures and social distancing policies have exacerbated mental health issues, particularly among the elderly and individuals with preexisting conditions [14]. In the context of accelerated globalization and urbanization, the speed and scope of infectious disease transmission have significantly increased, underscoring the urgent need for global cooperation and coordinated responses [15].

Understanding and analyzing the global burden of vIDPs is crucial for formulating effective public health policies, optimizing resource allocation, and enhancing disease prevention and control capabilities. However, comprehensive assessments of the long-term trends and risk factors associated with the burden of vIDPs remain insufficient, necessitating further in-depth research. We aim to systematically analyze the global burden of vIDPs in 2021 based on the Global Burden of Diseases, Injuries, and Risk Factors Study 2021 (GBD 2021) data. It seeks to evaluate the distribution and trends of vIDPs among populations and regional characteristics, as well as to explore the primary factors influencing the transmission of vIDPs. The objective is to elucidate the patterns of the global burden of vIDPs, thereby providing scientific evidence to guide public health decision-making.

## Methods

### Data sources

We obtained data on rabies, dengue, acute hepatitis, HIV/AIDS, EVD, and COVID-19 from the GBD 2021 using the Global Health Data Exchange (GHDx) results tool. The GBD 2021 provides new demographic estimates for 204 countries and territories and 811 additional subnational locations from 1950 to 2021 [16]. Specifically, we collected data for HIV/AIDS, acute hepatitis, dengue, and rabies from 1990 to 2021, while data for EVD did not cover the entire range from 1990 to 2021, and data for COVID-19 were only available for 2020 and 2021. In this report, we used the GBD results tool to extract estimates of deaths, prevalence, incidence, and disability-adjusted life years (DALYs), along with their 95% uncertainty intervals (UI), to measure the burden of vIDPs across regions and countries from 1990 to 2021. In addition, we collected the total percentage change for each indicator over the 1990–2021 year range.

The GBD 2021 also calculated the Socio-Demographic Index (SDI) to represent the combined level of social and economic development related to health in each region. The SDI is the geometric mean of the 0–1 index of the total fertility rate for women under 25 years old, the mean education level (years of schooling) for the population aged 15 and older, and the lag-distributed income per capita. The 204 countries in GBD 2021 are divided into five quintiles (low, low-middle, middle, high-middle, and high) based on their 2021 national-level SDI estimates.

### Data analysis

Age-standardized rates per 100,000 people were extracted from the GBD database. The age-standardized rate was calculated using the following formula:

$$\text{Age}\text{-}\text{standardized rate}=\frac{{\sum }_{i=1}^{N}{a}_{i}{W}_{i}}{{\sum }_{i=1}^{N}{W}_{i}}$$

where ${a}_{i}$ is the age-specific rate in the $i$th age group, and ${W}_{i}$ represents the number of people (or the weight) in the same age group among the GBD standard population. $N$ is the number of age groups. The 95% Uls were defined as the 25th and 975th values of the ordered 1000 draws.

Smoothing spline models were used to evaluate the relationship between the burdens of vlDPs and SDl for the 21 regions and 204 countries and territories. The expected values were determined through a calculation that takes into account the SDl and disease rates across all locations. We fitted smoothing splines using the Locally Weighted Scatterplot Smoothing method which automatically determines the degree, number, and location of nodes (knots) based on the data and the span parameter. Spearman correlation analysis was used to estimate the r indices and *P* values for the association of age-standardised rate with SDl. *P* < 0.05 was considered statistically significant.

All data analysis and mapping were performed using the statistical software R 3.0.3 (Lucent Technologies, Jasmine Mountain, USA).

## Results

### Global overview

**Fig. 1:**
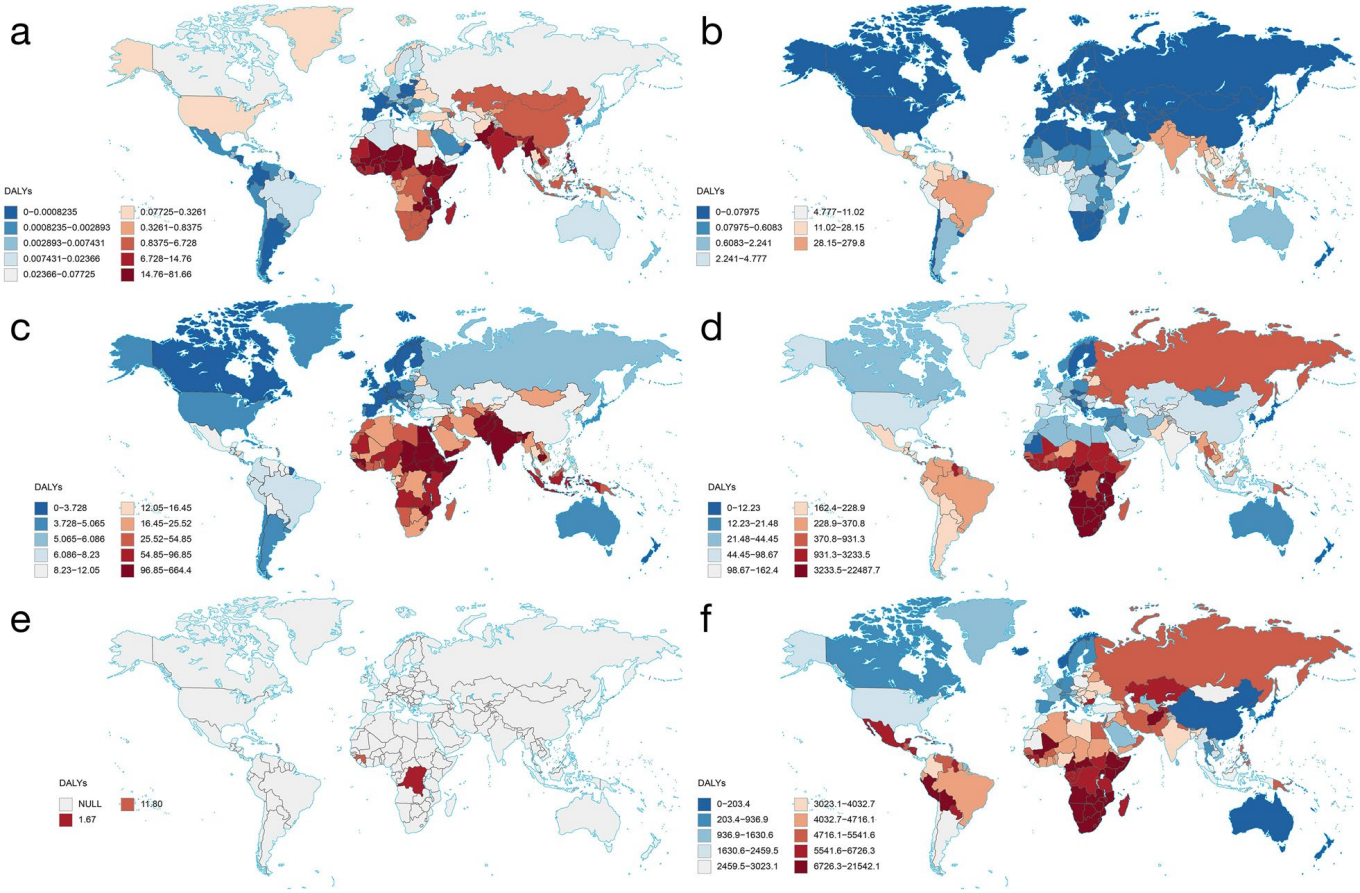
Global distribution of age-standardized DALY rates for six viral infectious diseases of poverty for all ages and both sexes in 2021 (**a** rabies, **b** dengue, **c** acute hepatitis, **d** HIV/AIDS, **e** EVD, and **f** COVID-19). *The rate is per 100,000 population. *DALY* disability-adjusted life year. Map approval number: GS(2024)3052

Globally, the six major vIDPs—rabies, dengue, acute hepatitis, HIV/AIDS, EVD, and COVID-19—resulted in significant health burdens (see Table 1). In 2021, the total deaths due to vIDPs were approximately 8.7 million, and the DALYs were approximately 259.2 million, accounting for 12.8% and 9.0% of the global all-cause totals, respectively. Table 1 summarizes the deaths, DALYs, incidence, and prevalence associated with each vIDPs. In 2021, COVID-19 was the most impactful disease among those listed, with approximately 7.9 million (95% UI: 7.5, 8.4) deaths and 212.0 million (95% UI: 197.9, 234.7) DALYs. This stark contrast with other diseases underscores the unprecedented global impact of the pandemic. Additionally, acute hepatitis had the second highest age-standardized incidence rate at 3411.5 (95% UI: 3201.8, 3631.3) per 100,000 population, and HIV/AIDS had the second highest age-standardized prevalence rate at 483.1 (95% UI: 459.0, 511.4) per 100,000 population. Compared to 1990, the deaths and DALYs of acute hepatitis in 2021 decreased, while the deaths and DALYs of HIV/AIDS increased. Moreover, the deaths, incidence and prevalence of dengue has increased, with deaths rising from 14,315 (95% UI: 11,103, 18,652) in 1990 to 29,076 (95% UI: 17,628, 38,981) in 2021. Remarkably, the burden of rabies has significantly reduced, with DALYs in 2021 decreasing by 58.4% (95% UI: 44.8%, 71.9%) compared to 1990. Although the incidence and prevalence rates of rabies are relatively low, its high mortality rate continues to have a significant impact on the global disease burden. In comparison, the burden of EVD remains relatively low, with 48 (95% UI: 39, 56) deaths and 2602 (95% UI: 2130, 3062) DALYs in 2021. Figure 1 illustrates the global distribution of age-standardized DALYs rate for these diseases in 2021.

**Table 1:** Global burden of six viral infectious diseases of poverty in 2021 and 1990, with percentage change (numbers and rates)

vIDPs	All ages number (95% UI)			Age-standardized rate (95% UI)		
	2021	1990	% change	2021	1990	% change
*Rabies*						
Deaths	10,084 (6016, 14,172)	21,806 (15,577, 28,435)	− 53.8 (− 66.5, − 40.5)	0.1 (0.1, 0.2)	0.4 (0.3, 0.5)	− 69.4 (− 77.6, − 61.0)
DALYs	569,550 (323,362, 828,522)	1,368,780 (978,542, 1,786,737)	− 58.4 (− 71.9, − 44.8)	7.5 (4.2, 11.0)	24.5 (17.5, 31.9)	− 69.4 (− 79.3, − 59.5)
Incidence	10,181 (6081, 14,293)	22,035 (15,732, 28,729)	− 53.8 (− 66.5, − 40.4)	0.1 (0.1, 0.2)	0.4 (0.3, 0.5)	− 69.4 (− 77.6, − 61.0)
Prevalence	391 (234, 549)	847 (605, 1104)	− 53.8 (− 66.5, − 40.4)	0.0 (0.0, 0.0)	0.0 (0.0, 0.0)	− 69.4 (− 77.6, − 61.0)
*Dengue*						
Deaths	29,076 (17,628, 38,981)	14,315 (11,103, 18,652)	103.1 (0.9, 225.6)	0.4 (0.2, 0.5)	0.3 (0.2, 0.3)	40.5 (− 26.6, 114.4)
DALYs	2,076,525 (1,056,228, 3,130,718)	1,248,669 (876,050, 1,552,997)	66.3 (− 22.5, 153.3)	27.8 (14.2, 41.7)	21.6 (15.1, 26.9)	28.3 (− 38.0, 91.7)
Incidence	58,964,185 (15,473,439, 106,885,036)	26,447,129 (3,933,186, 5,1891,942)	123.0 (75.3, 378.1)	752.0 (196.3, 1363.4)	481.8 (70.8, 946.3)	56.1 (22.6, 237.9)
Prevalence	3,517,384 (928,244, 6,430,040)	1,577,834 (234,723, 3,169,007)	122.9 (76.2, 374.5)	44.9 (11.8, 82.1)	28.7 (4.2, 57.8)	56.1 (23.4, 236.2)
*Acute hepatitis*						
Deaths	71,846 (58,045, 92,908)	160,352 (139,545, 180,112)	− 55.2 (− 63.0, − 44.2)	0.9 (0.7, 1.2)	3.2 (2.8, 3.6)	− 71.1 (− 76.3, − 63.8)
DALYs	4,228,048 (3,342,182, 5,559,113)	9,841,371 (8,674,224, 11,047,444)	− 57.0 (− 66.2, − 44.2)	56.7 (44.1, 75.6)	177.4 (156.2, 199.3)	− 68.0 (− 74.9, − 58.5)
Incidence	250,774,458 (234,361,297, 268,115,472)	266,388,078 (243,212,898, 289,980,844)	− 5.9 (− 12.1, 0.4)	3411.5 (3201.8, 3631.3)	4660.0 (4213.0, 5125.1)	− 26.8 (− 31.8, − 21.5)
Prevalence	21,553,992 (19,890,335, 23,361,736)	20,248,085 (18,524,954, 22,021,321)	6.4 (− 0.8, 13.8)	290.6 (269.5, 312.7)	352.3 (320.5, 383.7)	− 17.5 (− 23.2, − 12.2)
*HIV/AIDS*						
Deaths	718,079 (669,271, 785,447)	305,945 (234,538, 404,774)	134.7 (88.6, 204.7)	8.7 (8.1, 9.6)	5.9 (4.5, 7.8)	47.5 (18.6, 91.2)
DALYs	40,266,792 (37,138,092, 44,796,387)	18,673,403 (14,782,537, 24,225,400)	115.6 (78.1, 168.5)	496.4 (456.1, 554.2)	346.9 (273.1, 451.8)	43.1 (19.1, 77.8)
Incidence	1,645,333 (1,484,721, 1,822,432)	2,008,916 (1,843,346, 2,181,339)	− 18.1 (− 27.1, − 7.3)	20.8 (18.8, 23.0)	36.5 (33.5, 39.8)	− 43.2 (− 49.7, − 35.4)
Prevalence	40,036,936 (38,036,249, 42,372,669)	7,934,077 (7,417,414, 8,495,410)	404.6 (368.4, 446.5)	483.1 (459.0, 511.4)	149.2 (139.6, 159.6)	223.8 (200.5, 251.3)
*EVD*						
Deaths	48 (39, 56)	NA	NA	0.0 (0.0, 0.0)	NA	NA
DALYs	2602 (2130, 3062)	NA	NA	0.0 (0.0, 0.0)	NA	NA
Incidence	94 (82, 106)	NA	NA	0.0 (0.0, 0.0)	NA	NA
Prevalence	62 (18, 163)	NA	NA	0.0 (0.0, 0.0)	NA	NA
*COVID-19*						
Deaths	7,887,554 (7,507,141, 8,403,575)	NA	NA	94.0 (89.5, 100.0)	NA	NA
DALYs	212,009,596 (197,942,419, 234,674,235)	NA	NA	2500.7 (2330.2, 2778.2)	NA	NA
Incidence	2,279,717,768 (2,181,359,056, 2,372,460,275)	NA	NA	28,955.3 (27,708.3, 30,143.2)	NA	NA
Prevalence	152,308,807 (117,582,720, 214,647,715)	NA	NA	1911.7 (1479.0, 2688.6)	NA	NA

*vIDPs* viral infectious diseases of poverty; *UI* uncertainty intervals; *DALYs* disability-adjusted life years; *EVD* Ebola virus disease; *NA* Not applicable

### Trends by global and different SDI levels

**Fig. 2:**
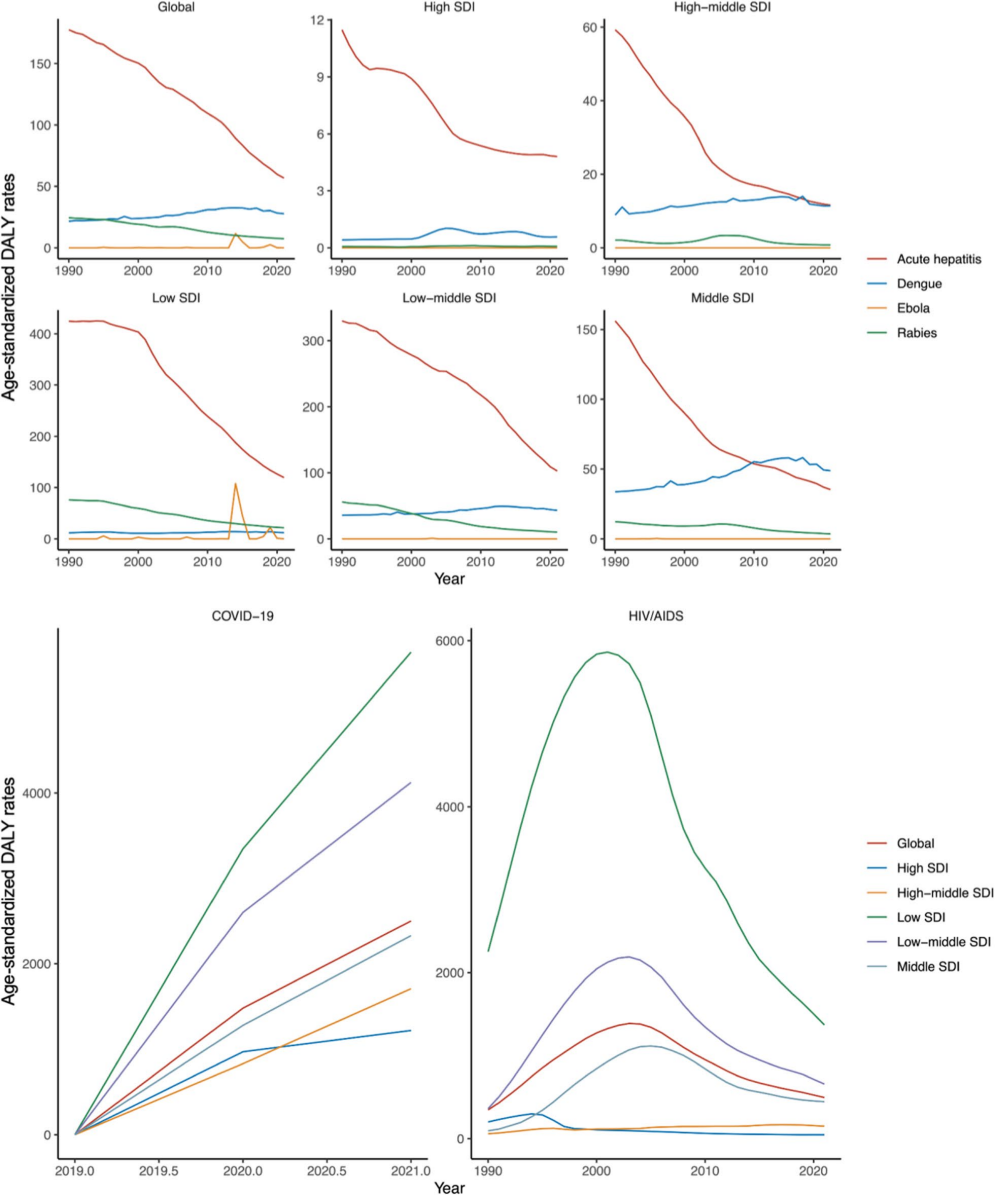
Trends in age-standardized DALY rates of six viral infectious diseases of poverty globally and by different SDI levels, 1990–2021. *The rate is per 100,000 population. *DALY* disability-adjusted life year; *SDI* Socio-Demographic Index

The trends depicted in Fig. 2 emphasize the dynamic and diverse impact of vIDPs globally and across different SDI contexts. From 1990 to 2021, the age-standardized DALY rate for HIV/AIDS peaked around 2003 at 1387.9 (95% UI: 1156.5, 1650.2) per 100,000 population globally, followed by a downward trend. However, in low SDI regions, HIV/AIDS continues to impose a significant burden, with age-standardized DALY rate remaining considerably higher than in other regions. COVID-19, which emerged at the end of 2019, rapidly escalated to become the leading cause of DALYs among the six diseases by 2021, demonstrating widespread impact irrespective of economic status. The age-standardized DALY rate for dengue initially increased, peaking in 2014 at 32.5 (95% UI: 20.5, 46.3) per 100,000 population. However, by 2021, it had only decreased to levels comparable to those around 2007, at 27.8 (95% UI: 14.2, 41.7) per 100,000 population. Particularly in low-middle and middle SDI regions, the disease continues to pose substantial challenges, with a considerable burden remaining as of 2021. Acute hepatitis and rabies exhibited relatively stable declining trends globally and across different SDI levels, with global reductions of approximately 68.0% (95% UI: 58.5%, 74.9%) for acute hepatitis and 69.4% (95% UI: 59.5%, 79.3%) for rabies in 2021 compared to 1990. Despite contributing to lower overall age-standardized DALY rates compared to other diseases, EVD showed notable spikes during specific outbreaks, particularly in low SDI regions.

### Age and gender differences

**Fig. 3:**
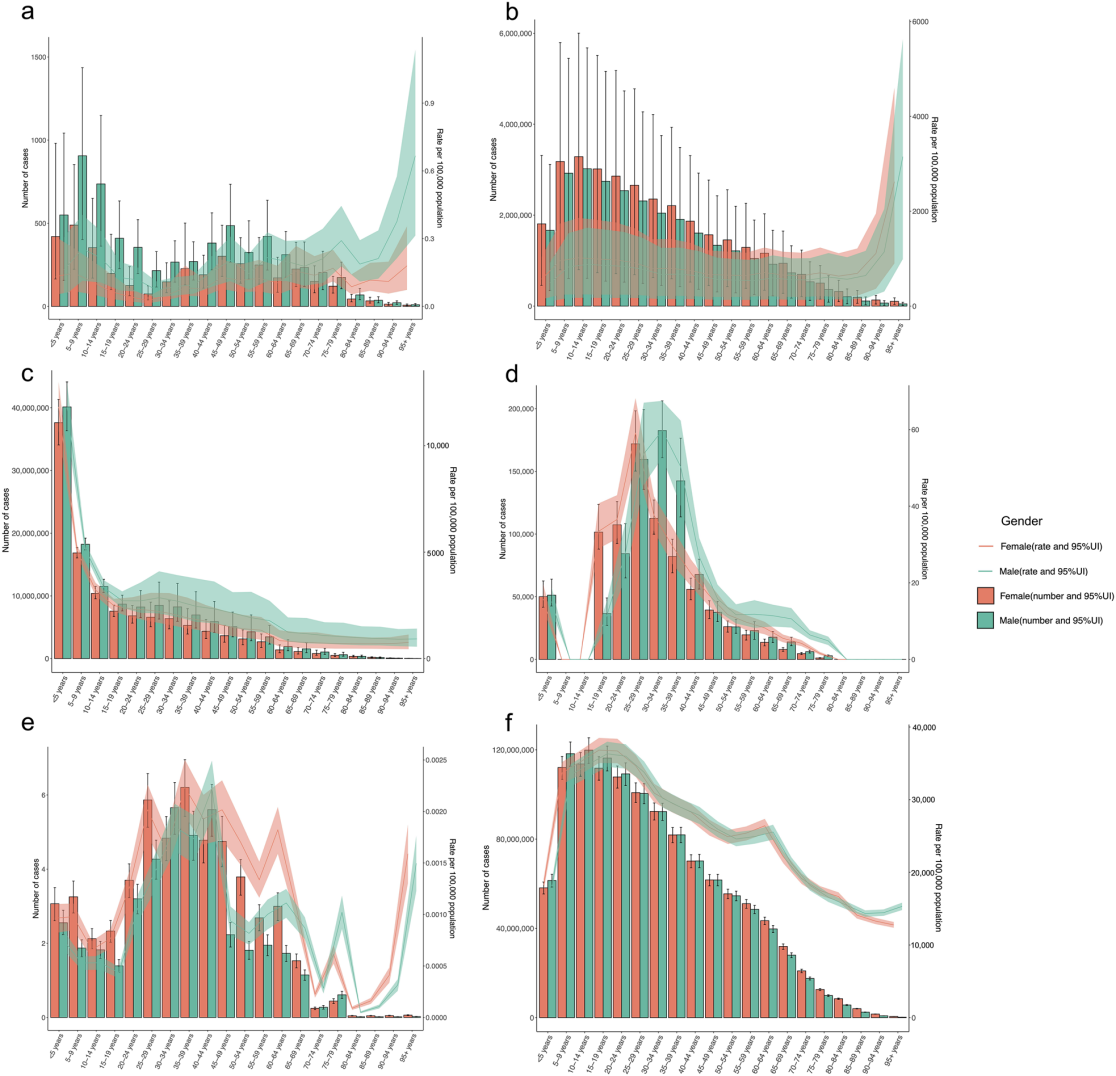
Age and gender differences in incidence rates and number of cases of six viral infectious diseases of poverty in 2021 (**a**: rabies, **b**: dengue, **c**: acute hepatitis, **d** HIV/AIDS, **e**: EVD, and **f**: COVID-19)

The incidence rates and number of cases of vIDPs in 2021 exhibit distinct patterns across different age groups and genders, as shown in Fig. 3.

For rabies, both the incidence rates and number of cases are relatively high among children and adolescents. The incidence rate is highest among the elderly, especially those aged 95 and above, with an incidence rate of 0.3 (95% UI: 0.2, 0.5) per 100,000 population. There is no statistically significant difference in the incidence rates and number of cases between males and females, with the total number of male cases being 6381 (95% UI: 3528, 8964) compared to 3800 (95% UI: 2202, 6184) in females.

For dengue, the incidence reaches its peak in the 10–14 years age group, with 6.3 million cases (95% UI: 1.5, 11.7). The incidence rate is also very high among those aged 95 and above, at 2768.8 (95% UI: 1012.7, 4885.7) per 100,000 population. Also, there is no statistically significant difference between males and females, with the total number of male cases being 27.3 million (95% UI: 6.0, 50.3) and 31.6 million (95% UI: 8.5, 56.6) in females.

Acute hepatitis shows a significantly higher risk of incidence among children and adolescents, particularly in children under 5 years old, with 77.8 million cases (95% UI: 70.4, 85.4) and an incidence rate of 11,815.5 (95% UI: 10,696.9, 12,969.7) per 100,000 population. Overall, the incidence rate among males in 2021 was significantly higher than among females, with males accounting for 3408.0 (95% UI: 3165.8, 3673.0) per 100,000 population and females accounting for 2946.1 (95% UI: 2754.0, 3153.8) per 100,000 population.

Young adults are the most affected by HIV/AIDS, with the incidence rate peaking in females at ages 25–29 years at 59.1 (95% UI: 51.6, 68.2) per 100,000 population, while the incidence rate for males peaks slightly later at ages 30–34 years at 59.8 (95% UI: 52.6, 67.5) per 100,000 population. Additionally, children under 5 years are another significant group affected by HIV/AIDS, with an incidence rate of 15.4 (95% UI: 12.8, 19.2) per 100,000 population. Among those aged 15–24, the incidence rate is significantly higher in females than in males. However, in the age groups 30–39 and 65–79, the incidence rate is significantly higher in males than in females.

The incidence of EVD is relatively low across all age groups, with a minor peak in children under 10 years and a more significant peak at ages 35–39 years, reaching an incidence rate of 2.0 (95% UI: 1.7, 2.2) per 100,000,000 population. The incidence rate decreases after age 40 but shows a slight increase again among those aged 75 and above. Overall, the incidence rate among females in 2021 was significantly higher than among males, with females accounting for 1.0 (95% UI: 0.9, 1.2) per 100,000,000 population and males accounting for 1.3 (95% UI: 1.2, 1.5) per 100,000,000 population.

For COVID-19, the highest risk of infection is observed among adolescents and young adults, particularly in the 15–19 years age group, where the incidence rate peaks at 36,557.6 (95% UI: 34,763.1, 38,238.8) per 100,000 population. The incidence rate decreases with age but shows an increase again among those aged 55 and above. This indicates that older adults face a higher risk of infection during the COVID-19 pandemic, possibly due to declining immunity and underlying health issues. Also, there is no statistically significant difference between males and females, with males accounting for 28,770.2 (95% UI: 27,525.9, 29,956.0) per 100,000 population and females accounting for 29,008.3 (95% UI: 27,764.9, 30,172.8) per 100,000 population.

### Regional differences

**Fig. 4:**
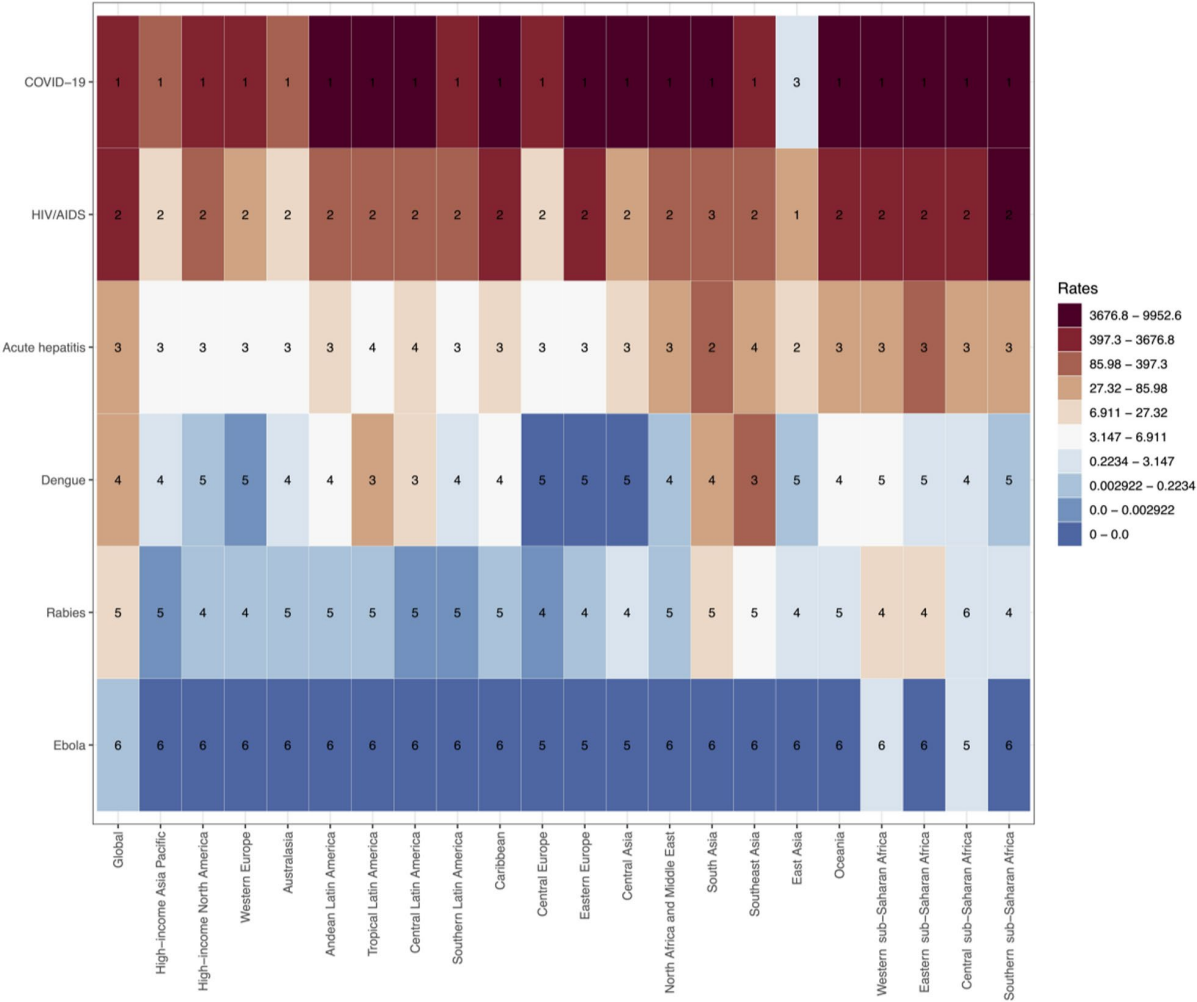
Heatmap of age-standardized DALY rates for six viral infectious diseases of poverty by 21 regions in 2021. *The rate is per 100,000 population. *DALY* disability-adjusted life year

In 2021, in most regions, the global burden was highest for COVID-19, followed by HIV/AIDS, acute hepatitis, dengue, rabies, and lowest for EVD, as shown in Fig. 4. COVID-19 caused the highest DALYs in nearly all regions worldwide, underscoring its extensive impact. However, in East Asia, it ranked third with age-standardized DALY rate of 2.8 (95% UI: 0.8, 9.2) per 100,000 population. Sub-Saharan Africa bears the heaviest burden of HIV/AIDS, with DALY rates reaching 9615.8 (95% UI: 9003.0, 10,270.0) per 100,000 population, highlighting severe public health challenges. South Asia and Eastern sub-Saharan Africa show high DALY rates for acute hepatitis, with 140.3 (95% UI: 103.2, 197.4) and 129.0 (95% UI: 104.3, 163.0) per 100,000 population respectively. Southeast Asia exhibits a high DALY rate for dengue at 147.0 (95% UI: 95.3, 201.0) per 100,000 population. Rabies imposes a substantial burden in Eastern and Western sub-Saharan Africa, with DALY rates of 26.7 (95% UI: 10.3, 59.7) and 19.8 (95% UI: 7.9, 31.4) per 100,000 population, respectively. Central sub-Saharan Africa faces a significant impact from EVD, with a DALY rate of 1.1 (95% UI: 0.9, 1.3) per 100,000 population.

Table 2 presents the progress and ongoing challenges in managing the burden of major vIDPs across different regions over the past three decades. In both 1990 and 2021, Africa remained the region most severely affected by HIV/AIDS. Notably, while the burden in Eastern sub-Saharan Africa has decreased, it has significantly increased in Southern sub-Saharan Africa, with a percentage change of 225.0 (95% UI: 133.4, 343.9), and in Western sub-Saharan Africa, with a percentage change of 58.6 (95% UI: 18.5, 117.7). Additionally, the burden of HIV/AIDS has also markedly increased in Asia and Oceania. In contrast, the burden of acute hepatitis in 2021 showed a significant decline across all 21 regions globally compared to 1990, with a percentage change of − 68.0 (95% UI: − 74.9, − 58.5) globally. Meanwhile, regions such as Southeast Asia, South Asia, and Tropical Latin America have consistently been hotspots for dengue, continuing to exhibit high age-standardized DALY rates in both 1990 and 2021. Moreover, in high-income North America, while the overall burden of dengue remains relatively low, the DALY rate in 2021 showed a significant increase compared to 1990, with a percentage change of 427.1 (95% UI: 102.1, 1029.3). This rise suggests emerging threats and the need for vigilant public health measures to prevent outbreaks. Finally, globally, the DALY rate for rabies decreased by 69.4% (95% UI: 59.5%, 79.3%). However, in regions such as high-income North America, Western Europe, and Australasia, the burden of rabies increased significantly compared to 1990. This upward trend highlights the necessity for continued and enhanced rabies prevention efforts.

**Table 2:** Age-standardized DALY rates and percentage change for major viral infectious diseases of poverty in 1990 and 2021

vIDPs	Age-standardized DALY rates 2021 (95% UI)	Age-standardized DALY rates 1990 (95% UI)	% Change (95% UI)
*HIV/AIDS*			
Global	496.4 (456.1, 554.2)	346.9 (273.1, 451.8)	43.1 (19.1, 77.8)
High-income Asia Pacific	7.6 (6.8, 8.7)	3.1 (2.8, 3.4)	149.5 (131.4, 174.6)
High-income North America	88.5 (72.9, 108.6)	491.6 (478.6, 508.6)	− 82.0 (− 84.8, − 78.7)
Western Europe	37.6 (33.0, 43.6)	138.8 (135.7, 142.3)	− 72.9 (− 75.7, − 69.1)
Australasia	13.3 (11.3, 16.2)	103.5 (101.3, 106.6)	− 87.1 (− 88.8, − 84.7)
Andean Latin America	236.3 (222.1, 253.4)	67.0 (64.5, 71.3)	252.7 (224.5, 279.3)
Tropical Latin America	300.9 (290.0, 315.1)	300.9 (296.3, 307.5)	0.0 (− 2.4, 2.7)
Central Latin America	243.0 (236.5, 251.4)	167.1 (165.2, 169.6)	45.4 (42.9, 48.6)
Southern Latin America	177.4 (168.4, 190.6)	87.5 (84.1, 91.4)	102.7 (96.7, 110.5)
Caribbean	831.0 (677.7, 1042.1)	1325.4 (941.5, 1886.4)	− 37.3 (− 53.3, − 16.7)
Central Europe	23.3 (22.3, 24.7)	25.2 (24.8, 25.6)	− 7.3 (− 10.4, − 2.9)
Eastern Europe	628.4 (605.8, 656.9)	115.0 (113.1, 118.3)	446.3 (433.7, 461.6)
Central Asia	78.4 (76.2, 81.2)	35.9 (35.6, 36.4)	118.0 (113.3, 124.5)
North Africa and Middle East	87.5 (56.0, 175.2)	13.1 (9.4, 24.3)	565.5 (175.6, 1553.4)
South Asia	129.9 (97.3, 200.7)	1.9 (1.0, 3.4)	6759.2 (3479.0, 13,428.2)
Southeast Asia	233.4 (216.7, 260.7)	138.4 (135.7, 141.4)	68.6 (56.5, 86.8)
East Asia	82.4 (59.6, 108.5)	6.6 (1.3, 10.5)	1151.7 (547.6, 7869.3)
Oceania	421.4 (301.1, 711.0)	21.0 (19.6, 23.0)	1910.9 (1335.7, 3242.4)
Western sub-Saharan Africa	2103.4 (1790.9, 2539.7)	1326.0 (887.1, 1999.2)	58.6 (18.5, 117.7)
Eastern sub-Saharan Africa	3372.1 (3034.8, 3767.8)	5426.6 (3753.3, 7793.1)	− 37.9 (− 54.7, − 7.6)
Central sub-Saharan Africa	1808.3 (1472.5, 2305.8)	2381.7 (1638.2, 3488.6)	− 24.1 (− 47.6, 9.2)
Southern sub-Saharan Africa	9615.8 (9003.0, 10,270.0)	2958.4 (2190.3, 4165.6)	225.0 (133.4, 343.9)
*Acute hepatitis*			
Global	56.7 (44.1, 75.6)	177.4 (156.2, 199.3)	− 68.0 (− 74.9, − 58.5)
High-income Asia Pacific	5.1 (3.7, 7.0)	10.0 (8.2, 12.2)	− 49.2 (− 58.5, − 40.7)
High-income North America	4.0 (3.0, 5.3)	5.2 (4.2, 6.5)	− 23.6 (− 29.9, − 17.8)
Western Europe	3.5 (2.5, 4.8)	4.5 (3.4, 6.0)	− 22.0 (− 27.7, − 16.4)
Australasia	3.9 (2.6, 5.6)	4.5 (3.2, 6.2)	− 12.6 (− 22.3, − 2.7)
Andean Latin America	7.0 (5.3, 9.1)	13.7 (11.2, 16.8)	− 49.1 (− 60.6, − 37.3)
Tropical Latin America	6.8 (5.3, 8.8)	15.1 (13.1, 18.1)	− 54.6 (− 62.3, − 47.7)
Central Latin America	8.6 (6.9, 10.8)	17.2 (15.0, 19.8)	− 49.8 (− 57.2, − 40.8)
Southern Latin America	4.9 (3.4, 6.6)	9.3 (7.2, 13.5)	− 47.9 (− 65.7, − 35.9)
Caribbean	13.7 (9.5, 18.8)	21.4 (14.7, 32.4)	− 36.3 (− 54.5, − 10.0)
Central Europe	5.2 (3.7, 7.0)	8.5 (6.8, 10.7)	− 39.3 (− 47.4, − 31.3)
Eastern Europe	6.7 (5.3, 8.7)	17.7 (15.9, 19.9)	− 62.3 (− 68.7, − 55.8)
Central Asia	22.9 (17.6, 30.5)	186.3 (166.7, 210.3)	− 87.7 (− 90.8, − 83.4)
North Africa and Middle East	57.1 (42.9, 77.8)	274.1 (200.8, 355.0)	− 79.2 (− 84.5, − 72.4)
South Asia	140.3 (103.2, 197.3)	374.8 (320.6, 435.0)	− 62.6 (− 75.4, − 42.7)
Southeast Asia	41.0 (34.8, 49.4)	155.9 (116.5, 206.7)	− 73.7 (− 80.8, − 62.0)
East Asia	11.8 (9.3, 15.0)	148.8 (127.0, 173.8)	− 92.0 (− 94.1, − 89.7)
Oceania	36.0 (18.9, 58.2)	130.1 (68.9, 204.6)	− 72.3 (− 80.6, − 56.1)
Western sub-Saharan Africa	71.1 (48.1, 96.5)	459.0 (236.0, 701.4)	− 84.5 (− 88.9, − 76.3)
Eastern sub-Saharan Africa	129.0 (104.3, 163.0)	292.6 (240.0, 371.5)	− 55.9 (− 65.1, − 43.7)
Central sub-Saharan Africa	37.3 (23.5, 52.3)	199.5 (118.2, 316.7)	− 81.3 (− 87.3, − 70.4)
Southern sub-Saharan Africa	34.4 (23.5, 50.4)	65.7 (36.6, 96.9)	− 47.7 (− 61.3, − 25.5)
*Dengue*			
Global	27.8 (14.2, 41.7)	21.6 (15.1, 26.9)	28.3 (− 38.0, 91.7)
High-income Asia Pacific	2.9 (0.5, 7.4)	1.3 (0.2, 3.5)	122.6 (− 21.0, 1012.3)
High-income North America	0.0 (0.0, 0.0)	0.0 (0.0, 0.0)	427.1 (102.1, 1029.3)
Western Europe	0.0 (0.0, 0.0)	0.0 (0.0, 0.0)	− 99.0 (− 99.7, − 97.3)
Australasia	0.6 (0.2, 1.6)	0.3 (0.0, 1.0)	107.8 (− 59.5, 4136.7)
Andean Latin America	6.6 (2.8, 12.3)	3.6 (0.7, 8.9)	83.0 (− 15.4, 909.9)
Tropical Latin America	63.8 (22.4, 136.0)	43.6 (3.3, 113.6)	46.4 (− 17.1, 834.2)
Central Latin America	17.5 (9.9, 27.6)	8.3 (2.4, 17.7)	112.3 (31.1, 515.1)
Southern Latin America	1.2 (0.2, 3.3)	0.8 (0.0, 3.0)	50.6 (− 50.9, 3544.6)
Caribbean	5.8 (1.2–15.5)	5.0 (0.8, 14.8)	16.0 (− 41.6, 206.9)
Central Europe	0.0 (0.0, 0.0)	0.0 (0.0, 0.0)	NA
Eastern Europe	0.0 (0.0, 0.0)	0.0 (0.0, 0.0)	NA
Central Asia	0.0 (0.0, 0.0)	0.0 (0.0, 0.0)	NA
North Africa and Middle East	0.2 (0.1, 0.4)	0.3 (0.2, 0.4)	− 26.8 (− 67.5, 85.6)
South Asia	53.5 (19.5, 91.9)	35.8 (18.0, 57.4)	49.4 (− 14.5, 103.3)
Southeast Asia	147.0 (95.3, 201.0)	135.0 (88.8, 204.7)	8.9 (− 49.5, 91.0)
East Asia	0.1 (0.0, 0.2)	0.3 (0.2, 0.5)	− 72.2 (− 84.3, − 53.4)
Oceania	6.0 (2.7, 11.9)	13.4 (8.6, 20.8)	− 55.0 (− 73.2, − 23.7)
Western sub-Saharan Africa	4.9 (0.2, 17.0)	3.7 (0.0, 14.3)	33.9 (− 45.5, 3259.1)
Eastern sub-Saharan Africa	1.3 (0.2, 4.1)	8.2 (0.7, 21.5)	− 84.4 (− 98.6, 245.6)
Central sub-Saharan Africa	1.9 (0.1, 10.3)	1.4 (0.0, 8.4)	40.2 (− 84.1, 1135.1)
Southern sub-Saharan Africa	0.0 (0.0, 0.1)	0.0 (0.0–0.1)	− 2.4 (− 90.8, 923.1)
*Rabies*			
Global	7.5 (4.2, 11.0)	24.5 (17.5, 31.9)	− 69.4 (− 79.3, − 59.5)
High-income Asia Pacific	0.0 (0.0, 0.0)	0.0 (0.0, 0.0)	− 92.7 (− 93.7, − 91.6)
High-income North America	0.1 (0.1, 0.1)	0.0 (0.0, 0.0)	162.3 (131.5, 195.6)
Western Europe	0.0 (0.0, 0.0)	0.0 (0.0, 0.0)	113.0 (84.3, 145.0)
Australasia	0.0 (0.0, 0.0)	0.0 (0.0, 0.0)	76.1 (9.6, 191.2)
Andean Latin America	0.0 (0.0, 0.0)	2.6 (2.0, 3.1)	− 99.9 (− 99.9, − 99.8)
Tropical Latin America	0.0 (0.0, 0.0)	3.1 (2.1, 4.3)	− 99.7 (− 99.8, − 99.6)
Central Latin America	0.0 (0.0, 0.0)	2.3 (2.1, 2.5)	− 99.9 (− 99.9, − 99.9)
Southern Latin America	0.0 (0.0, 0.0)	0.0 (0.0, 0.0)	− 49.3 (− 58.9, − 36.8)
Caribbean	0.0 (0.0, 0.1)	0.0 (0.0, 0.1)	− 6.6 (− 54.7, 93.3)
Central Europe	0.0 (0.0, 0.0)	0.1 (0.1, 0.1)	− 98.3 (− 98.8, − 97.6)
Eastern Europe	0.1 (0.1, 0.1)	0.3 (0.3, 0.3)	− 72.6 (− 77.2, − 66.3)
Central Asia	0.5 (0.3, 0.9)	1.2 (0.6, 2.3)	− 53.9 (− 75.5, − 14.7)
North Africa and Middle East	0.1 (0.1, 0.2)	1.6 (0.8, 2.4)	− 91.4 (− 94.7, − 85.4)
South Asia	14.2 (9.1, 20.2)	79.9 (59.2, 99.4)	− 82.2 (− 86.4, − 76.5)
Southeast Asia	5.4 (3.1, 8.2)	18.6 (14.1, 25.4)	− 71.1 (− 82.0, − 57.7)
East Asia	1.8 (1.0, 2.8)	6.0 (3.5, 8.4)	− 69.8 (− 79.9, − 57.0)
Oceania	0.6 (0.1, 1.6)	0.7 (0.2, 2.0)	− 13.1 (− 54.3, 66.8)
Western sub-Saharan Africa	19.8 (7.9, 31.4)	35.8 (16.3, 62.7)	− 44.8 (− 69.5, − 11.4)
Eastern sub-Saharan Africa	26.7 (10.3, 59.7)	76.6 (35.4, 167.6)	− 65.2 (− 81.0, − 41.8)
Central sub-Saharan Africa	0.9 (0.2, 2.5)	1.7 (0.3, 5.2)	− 46.6 (− 70.2, − 7.2)
Southern sub-Saharan Africa	2.2 (1.3, 3.7)	3.2 (1.8, 5.0)	− 30.6 (− 56.5, 16.6)

### Relationship between vIDPs and SDI

**Fig. 5:**
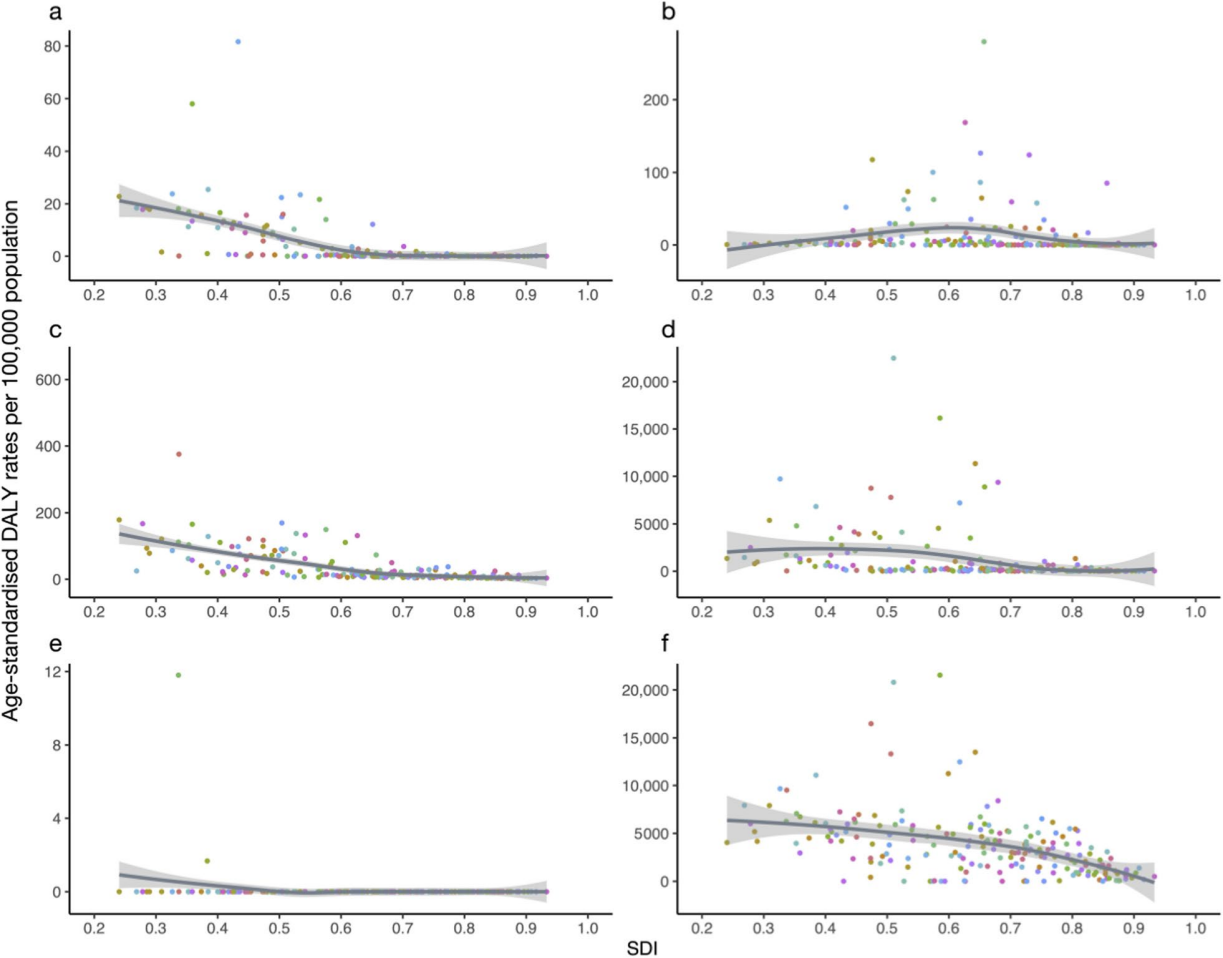
Relationship between SDI and age-standardized DALY rates for six viral infectious diseases of poverty in 2021 (**a**: rabies, **b**: dengue, **c**: acute hepatitis, **d**: HIV/AIDS, **e**: EVD, and **f**: COVID-19). *SDI: Socio-Demographic Index; DALY: disability-adjusted life year

The analysis reveals that the age-standardized DALY rates for vIDPs are all significantly negatively correlated with SDI, as shown in Fig. 5. Notably, there is a strong negative correlation between the DALY rates of acute hepatitis and SDI (r = − 0.8, *P* < 0.0001). Low SDI countries like Afghanistan show a significant disease burden, with DALY rates of 375.4 (95% UI: 251.6, 543.7) per 100,000 population. Similarly, rabies DALY rates also demonstrate a strong negative correlation with SDI (r = − 0.7, *P* < 0.0001). High SDI regions, such as high-income North America, Western Europe, and Australia, have DALY rates close to zero. In contrast, countries like Chad experience substantial disease burdens for both acute hepatitis and rabies, with acute hepatitis DALY rates of 178.9 (95% UI: 124.3, 254.0) per 100,000 population and rabies DALY rates of 22.8 (95% UI: 3.3, 59.4) per 100,000 population.

Furthermore, the analysis shows moderate negative correlations between the DALY rates of HIV/AIDS (r = − 0.6, *P* < 0.0001), COVID-19 (r = − 0.5, *P* < 0.0001), and dengue (r = − 0.4, *P* < 0.0001) and SDI. In regions with low SDI, such as Southern Africa, the burden of HIV/AIDS and COVID-19 remains high. For example, Malawi has HIV/AIDS and COVID-19 DALY rates of 6839.4 (95% UI: 5900.8, 8128.0) and 11,072.5 (95% UI: 9592.5, 12,394.8) per 100,000 population, respectively. Similarly, South-East Asia experiences high dengue DALY rates, with Indonesia having DALY rates of 279.8 (95% UI: 170.9, 404.4) per 100,000 population. In contrast, EVD shows a relatively weaker but still statistically significant negative correlation with SDI (r = − 0.2, *P* < 0.005).

## Discussion

This study is a comprehensive report of the global burden of vIDPs. Based on the 2021 GBD data, the results highlight the disproportionate impact of these diseases on global health, particularly emphasizing the overwhelming burden of COVID-19, the persistent challenges posed by HIV/AIDS, the rising incidence of dengue, and the significant, albeit more contained, impacts of rabies, acute hepatitis, and EVD. The analysis reveals marked regional, age, and gender disparities in the burden of these diseases, underscoring the need for targeted public health interventions.

From a global perspective, the overall burden of vIDPs has significantly increased by 2021, primarily due to the recent emergence and pandemic of COVID-19. It is evident that COVID-19 has become the leading cause of DALYs among the vIDPs analyzed. The pandemic has not only disrupted long-term gains in life expectancy but has also reversed the reduction in many leading causes of death [17]. Furthermore, it is estimated that COVID-19 may push over 100 million people back into extreme poverty, with countries such as India, Nigeria, and the Democratic Republic of Congo experiencing the most severe economic downturns [18]. This economic impact threatens to undermine the progress made in controlling various neglected tropical diseases. Excluding COVID-19, the total DALYs for the other five vIDPs have shown a slight increase in 2021 compared to 1990, primarily due to the persistent impact of dengue and HIV/AIDS. The burden of dengue has steadily increased over the past thirty years, driven by factors such as urbanization, climate change [19], and increased population mobility. Regions like Southeast Asia and South Asia remain particularly concerning, and the Americas have also seen a rapid rise in dengue cases [20]. Additionally, the ongoing high burden of HIV/AIDS, especially in sub-Saharan Africa, continues to pose significant public health challenges. Researchers have highlighted how HIV/AIDS and COVID-19 disproportionately affect women, the poor, and those with limited access to sustainable food and medicine, referring to these conditions as pandemics of inequality [21]. Moreover, the low incidence and prevalence rates of Ebola reflect effective containment of outbreaks but also underscore the necessity for vigilance and rapid response.

This study also underscores the significant regional disparities in the burden of vIDPs. Sub-Saharan Africa bears the heaviest burden of HIV/AIDS, reflected in its high DALY rates and severe public health challenges. In 2021, an estimated 67% of the 38.4 million people living with HIV globally were from sub-Saharan Africa [22]. A substantial proportion of households in this region incur catastrophic health expenditures when accessing medical services [23]. Southeast Asia and South Asia demonstrate notably high burdens of dengue and acute hepatitis, respectively. Predictions suggest that dengue incidence in Southeast Asia will peak this century [24]. Currently, the region is hyperendemic for multiple dengue virus serotypes/genotypes, necessitating control methods that align with the local epidemiological landscape [25]. Studies on acute viral hepatitis among children and adolescents in 2019 indicate that the highest incidence rates occur in sub-Saharan Africa, Oceania, South Asia, and Central Asia. In contrast, economically developed regions exhibit significantly lower incidence rates, approximately one-fourth of those in high-burden areas [26], which aligns with our findings. Furthermore, the regional distribution of the COVID-19 burden highlights the extensive impact of the pandemic across socio-economic domains globally. COVID-19's widespread influence has disrupted healthcare systems, economies, and social structures, affecting both high and low-income regions. It is estimated that at least 65 million people worldwide suffer from long COVID [27], and extensive research into its long-term effects is ongoing [28].

The incidence rates of vIDPs show significant differences across age and gender, underscoring the necessity for targeted public health interventions. Children and adolescents are more susceptible to diseases such as rabies and dengue, highlighting the urgent need for robust vaccination and prevention programs for these age groups. The increased incidence of acute hepatitis among young children emphasizes the importance of early immunization and improved sanitary conditions. Despite the severity of these issues, their impact on children and adolescents is often overlooked, with research predominantly focused on adult treatments [26]. Elderly populations are particularly vulnerable to diseases like COVID-19 and rabies, indicating the need for enhanced protective measures and timely medical care. Dengue incidence is slightly higher in females, peaking at ages 10–14, consistent with GBD 2017 findings [29]. The incidence rate of acute hepatitis is higher in males, aligning with analyses based on GBD 2019 [30]. However, the gender differences for hepatitis A, B, C, D, and E may vary [30] and are not elaborated here. GBD 2021 data indicate that the incidence rate of EVD is significantly higher in females. During EVD outbreaks in Central and West Africa, women were more affected, not only in terms of illness and mortality but also through a range of social impacts [31, 32]. In contrast, the gender differences in HIV/AIDS incidence rates vary across different age groups. Studies show that in high and upper-middle SDI regions, the burden of HIV/AIDS is higher in males than in females, potentially due to changing sexual behaviors, an increase in the number of men who have sex with men, and rising HIV infection rates [33].These insights call for age- and gender-specific health policies to effectively mitigate the burden of vIDPs and improve health outcomes.

The relationship between SDI and the burden of vIDPs reveals important insights. Regions with higher SDI typically exhibit lower DALY rates for vIDPs, reflecting the positive impact of socioeconomic development on public health. Related studies using 2019 disease burden data have also confirmed the correlation between socio-economic development and the burden of acute viral hepatitis (AVH), indicating that cost-effective AVH interventions should be guided by these findings [30]. Similarly, the strong negative correlation for rabies highlights the disparity in healthcare resources and the need for improved public health measures. Additionally, research shows a narrowing trend in the inequality of HIV/AIDS burden across countries, with significant reductions primarily in low-income nations [34]. Data indicates that the SDI demonstrates a consistent bell-shaped relationship with dengue's age-standardized incidence rate, age-standardized death rate, and age-standardized DALYs rate [20]. The relatively weaker negative correlation for EVD suggests that while socio-demographic factors are important, other elements such as outbreak control measures and healthcare responses are also crucial in managing Ebola. These findings emphasize the need for a comprehensive approach that considers a wide range of factors in addressing the burden of vIDPs.

To effectively mitigate the burden of vIDPs, a multifaceted approach to intervention is crucial. First and foremost, surveillance, forecasting, and response are essential components [35]. Robust monitoring systems and early warning mechanisms must be established to detect and respond to outbreaks promptly. This includes preparedness measures to ensure swift and efficient emergency responses [36]. Second, implementing One Health actions is vital, recognizing the interconnectedness of human, animal, and environmental health [37]. This necessitates multidisciplinary, multi-sectoral, and cross-regional cooperation to address the root causes of vIDPs comprehensively [38]. Third, enhancing health education is paramount. Persistent efforts to promote personal hygiene and public awareness can significantly reduce transmission rates [39]. Fourth, community-based interventions [40], including patriotic health campaigns and active community participation, play a pivotal role. Engaging local communities in health initiatives ensures better adherence to preventive measures and fosters a supportive environment for disease control. Last, strengthening scientific research is imperative. Current gaps in antiviral drugs, vaccines, diagnostic tools, and multi-point triggered surveillance systems highlight the urgent need for continued innovation and development in these areas [41–43]. Addressing these deficiencies through dedicated research efforts will enhance our capacity to prevent and control vIDPs effectively.

This study has several limitations, primarily stemming from the methodologies used in the GBD 2021 study [16, 44], which have been discussed in previous publications. Additionally, there are specific limitations to this paper, including: (1) although we have conducted a relatively comprehensive assessment of the burden of vIDPs, our estimates do not include some diseases, such as avian influenza, due to the lack of authoritative burden estimates for these diseases; (2) the analysis of risk factors in GBD 2021 is limited, particularly for the major vIDPs we focused on, due to insufficient data, which prevented their inclusion in our analysis. (3) Data availability is limited in certain regions, especially low-income countries, leading to uncertainty in the estimated results.

## Conclusions

This study provides the first comprehensive analysis of the global burden of six major vIDPs based on the GBD 2021 data. The findings highlight the significant impact of COVID-19, HIV/AIDS, dengue, rabies, acute hepatitis, and EVD on the global health. Addressing these diseases requires targeted public health interventions, tailored strategies, and international cooperation to reduce their burden and improve global health outcomes.

## Data Availability

The full study protocol and the datasets, are available, following manuscript publication, upon request from the corresponding author (Xiao-Xi Zhang, zhangxiaoxi@sjtu.edu.cn).

## Abbreviations

AVH: Acute viral hepatitis
COVID-19: Coronavirus disease 2019
DALYs: Disability-Adjusted Life Years
GBD: Global Burden of Disease
GHDx: Global Health Data Exchange
HIV: Human immunodeficiency virus
SDI: Socio-Demographic Index
UI: Uncertainty intervals
vIDPs: Viral infectious diseases of poverty
